# Ecophysiological Performance of Proteaceae Species From Southern South America Growing on Substrates Derived From Young Volcanic Materials

**DOI:** 10.3389/fpls.2021.636056

**Published:** 2021-02-19

**Authors:** M. Delgado, A. Zúñiga-Feest, M. Reyes-Díaz, P. J. Barra, S. Ruiz, A. Bertin-Benavides, S. Valle, M. Pereira, H. Lambers

**Affiliations:** ^1^Center of Plant, Soil Interaction and Natural Resources Biotechnology, Scientific and Technological Bioresource Nucleus (BIOREN), Universidad de La Frontera, Temuco, Chile; ^2^Laboratorio de Biología Vegetal, Instituto de Ciencias Ambientales y Evolutivas, Facultad de Ciencias, Universidad Austral de Chile, Valdivia, Chile; ^3^Centro de Investigación en Suelos Volcánicos (CISVo), Valdivia, Chile; ^4^Departamento de Ciencias Químicas y Recursos Naturales, Facultad de Ingeniería y Ciencias, Universidad de La Frontera, Temuco, Chile; ^5^Laboratorio de Epigenética Vegetal, Departamento de Silvicultura, Facultad de Ciencias Forestales, Universidad de Concepción, Concepción, Chile; ^6^Facultad de Ciencias Agrarias, Instituto de Ingeniería Agraria y Suelos, Universidad Austral de Chile, Valdivia, Chile; ^7^School of Biological Sciences, The University of Western Australia, Perth, WA, Australia

**Keywords:** carboxylates, cluster roots, colonization, nutrients, Proteaceae, volcanic substrate-soils

## Abstract

Southern South American Proteaceae thrive on young volcanic substrates, which are extremely low in plant-available phosphorus (P). Most Proteaceae exhibit a nutrient-acquisition strategy based on the release of carboxylates from specialized roots, named cluster roots (CR). Some Proteaceae colonize young volcanic substrates which has been related to CR functioning. However, physiological functioning of other Proteaceae on recent volcanic substrates is unknown. We conducted an experiment with seedlings of five Proteaceae (*Gevuina avellana*, *Embothrium coccineum*, *Lomatia hirsuta*, *L. ferruginea*, and *L. dentata*) grown in three volcanic materials. Two of them are substrates with very low nutrient concentrations, collected from the most recent deposits of the volcanoes Choshuenco and Calbuco (Chile). The other volcanic material corresponds to a developed soil that exhibits a high nutrient availability. We assessed morphological responses (i.e., height, biomass, and CR formation), seed and leaf macronutrient and micronutrient concentrations and carboxylates exuded by roots. The results show that *G. avellana* was less affected by nutrient availability of the volcanic substrate, probably because it had a greater nutrient content in its seeds and produced large CR exuding carboxylates that supported their initial growth. *Embothrium coccineum* exhibited greater total plant height and leaf P concentration than *Lomatia* species. In general, in all species leaf macronutrient concentrations were reduced on nutrient-poor volcanic substrates, while leaf micronutrient concentrations were highly variable depending on species and volcanic material. We conclude that Proteaceae from temperate rainforests differ in their capacity to grow and acquire nutrients from young and nutrient-poor volcanic substrates. The greater seed nutrient content, low nutrient requirements (only for *G. avellana*) and ability to mobilize nutrients help explain why *G. avellana* and *E. coccineum* are better colonizers of recent volcanic substrates than *Lomatia* species.

## Introduction

Temperate forest ecosystems of southern South America are a unique biome that is biogeographically isolated with a highly endemic flora (Quintero et al., 2014). These ecosystems are frequently affected by catastrophic disturbances such as volcanic eruptions and earthquakes that usually trigger orogenic uplift and landslides (Veblen and Ashton, 1978; Veblen et al., 1992, 1996), which lead to soil rejuvenation, leaving bare areas where the process of primary succession begins again. Following these catastrophic events, volcanic eruptions leave large parts of the landscape covered with rocks formed from lava flow, stones or sandy substrates, which are generally nutrient-poor and with almost no organic matter. Likewise, it is common to find volcanic soils in southern Chile containing large amounts of active aluminum (Al^3+^), oxides and hydroxides of iron (Fe) and Al, humus-Al/Fe complexes and amorphous and poorly crystallized minerals (e.g., allophane), which strongly sorb phosphorus (P) (Borie and Rubio, 2003; Matus et al., 2006; Borie et al., 2019). Therefore, volcanic soils may contain large amounts of total P, but with a very low availability for plants.

The colonization of nutrient-impoverished environments involves species with specialized root structures (e.g., cluster roots) or symbiotic associations (e.g., mycorrhizas, nitrogen (N)-fixing structures, or/and P-solubilizing bacteria) (Lambers et al., 2008). Among them, some species of the Proteaceae family frequently colonize young volcanic substrates (Delgado et al., 2018). Species belonging to this family have been extensively studied, because many of them inhabit extremely nutrient-poor soils in southwestern Australia and South Africa, and are highly efficient at both acquiring and utilizing nutrients, especially P (Lambers et al., 2015a). The main nutrient-acquisition strategy of Proteaceae involves the formation of cluster roots (CR), which are clusters of dense hairy rootlets growing in longitudinal rows along lateral roots (Purnell, 1960) that efficiently mobilize nutrients from the soil by actively releasing exudates (Lamont, 2003; Shane and Lambers, 2005a; Lambers et al., 2006). Carboxylates are the main root exudates and are involved in several key processes in the rhizosphere including nutrient acquisition and metal mobilization/detoxification (Jones, 1998; Ryan et al., 2001; Chen and Liao, 2016). Carboxylates have negative charges, allowing the complexation of metal cations and the displacement of anions such as phosphate, from the soil matrix. Additionally, carboxylates increase the availability of some micronutrients through their solubilizing and reducing capacity, for example, Mn^4+^ to Mn^2+^ and Fe^3+^ to Fe^2+^, which are the forms that are taken up by roots that use Strategy I (i.e., roots of dicots and non-graminaceous monocots that release reducing/chelating substances and increase the plasma membrane-bound reductase activity) (Marschner et al., 1986; Dinkelaker et al., 1995). These micronutrients, like others transition metal cations such as zinc (Zn^2+^), can also be mobilized by carboxylates at the root surface, where they are taken up by plasma membrane transporters with a low specificity (Lambers et al., 2015b). Thus, carboxylate exudation by CR (which occurs in large quantities compared with that of non-CR) is an adaptive trait allowing species to thrive in nutrient-poor soils.

Southern South America is inhabited by six Proteaceae species: *Embothrium coccineum* J. R. Forst. & G. Forst., *Lomatia hirsuta* Lam., *L. ferruginea* Cav. R. Br., *L. dentata* Ruiz et Pavon R. Br., *Orites myrtoidea* Poepp. & Endl., and *Gevuina avellana* Mol. (Donoso, 2006). These species may co-occur along their geographical range between 36 and 44°S. However, *O. myrtoidea*, has the narrowest distribution (35–38°S) and occurs only in the Andes Mountains (Hechenleitner et al., 2005). Additionally, *L. dentata* and *L. hirsuta* may occur a little further north (32°S) and *L. ferruginea* and *E. coccineum*, may occur much further south (50° and 56°S, respectively) (Steubing et al., 1983). In their natural habitat, these Proteaceae grow in a wide range of soil conditions (Souto et al., 2009; Delgado et al., 2018, 2019) and some of them, such as *E. coccineum*, *O. myrtoidea*, and *G. avellana*, can thrive in young volcanic substrates with very low nutrient availability (Donoso, 2006; Zúñiga-Feest et al., 2018; Ávila-Valdés et al., 2019; Zúñiga-Feest et al., 2020). Conversely, *Lomatia* species perform better in relatively more fertile soils. In fact, *Lomatia hirsuta* is a successful pioneer on landslides or after forest clearing, while *L. ferruginea* and *L. dentata* grow better under the shade of other trees growing on more developed soils (Donoso, 2006; Zúñiga-Feest et al., 2020).

The South American Proteaceae are considered outliers from the main centers of Proteaceae diversity, which are found in South western Australia and South Africa (Prance and Plana, 1998), both ancient landscapes with severely P-impoverished soils (Lambers et al., 2010). The South American Proteaceae differ from the Proteaceae of Southwest Australia and South Africa, because, in general, they have lower seed P concentrations (Groom and Lamont, 2010; Delgado et al., 2015b). This suggests that seedling establishment of southern South American Proteaceae depends more heavily on nutrients in the substrate than on nutrient reserves in their seeds. In fact, except for the larger-seeded *G. avellana* (354 mg dry weight seed^–1^), the southern South American Proteaceae produce small seeds (≤17 mg seed^–1^ dry weight) (Delgado et al., 2014), and form CR, even before they shed their cotyledons (e.g., ∼1 month-old *E. coccineum* seedlings; Delgado et al., 2015b). We propose that CR formation is a key strategy for nutrient uptake at very early life stages. However, the ability of these species to establish and thrive in soils with a low P availability such as recent volcanic substrates, is not fully understood.

Recently, Zúñiga-Feest et al. (2020) reported that *G. avellana* grows faster than *L. dentata* in nutrient-poor volcanic sand, but this trend is the opposite when these plants are grown on the same volcanic substrate, supplemented with a complete nutrient solution. This finding suggests that these species differ in their ability to grow in soils/substrates with different nutrient availability, possibly due to their different nutritional demand or nutrient-acquisition strategy. Although all Proteaceae from southern south America can grow on nutrient-poor volcanic soils, it is unknown how they adjust their growth, biomass allocation to CR, and carboxylate exudation when they grow on volcanic soil/substrate with different nutrient availability. In order to address this question, we conducted an experiment where seedlings of five Proteaceae (*E. coccineum*, *L. hirsuta*, *L. ferruginea*, *L. dentata*, and *G. avellana*) were grown in three volcanic materials with different nutrient availability. We hypothesized that the colonizing ability strongly depends on nutrient demand of the species and their ability to sustain growth under low-nutrient conditions. Specifically, we expected that the seedling performance of the only larger-seeded species, *G. avellana*, will be less affected by nutrient availability of the soil/substrate, because the higher nutrient content in its seeds will support their initial growth. Additionally, *E. coccineum*, the species reported as colonizer of volcanic substrates, will perform well in both nutrient-rich volcanic soil and young nutrient-poor substrates due to its greater nutrient-uptake capacity and faster rates of root carboxylate exudation. In contrast, species belonging to the genus *Lomatia*, will perform better in nutrient-rich soils, because they have greater nutritional demands. Therefore, the aim of this study was to assess the differences in seedling performance of five temperate rainforest Proteaceae and the morpho-physiological traits involved in soils/substrates with different nutrient availability, aiming to understand their differential colonizing ability under field conditions. Understanding how these species perform in different soil conditions will be key to support restoration activities with these native species and the information generated could be useful to extrapolate to other species that present similar morpho-physiological traits.

## Materials and Methods

### Sampling Sites

Three volcanic materials were collected from the localities of Ensenada (41° 10′52.48′′S – 72° 27′16.74′′W), Choshuenco (39° 33′ 12′′S – 72° 8′ 43.44′′W) and Experimental Station “Agropecuaria Austral” (ESAA) (39° 45′30′ – 73° 14′55′′W), Chile. At one extreme, substrates from the localities of Choshuenco and Ensenada correspond to recent volcanic deposits. Thus, from the Ensenada site, we collected deposits of volcanic sand from the last eruption of “Calbuco” volcano (on 23–24 April, 2015), and from the Choshuenco site, deposits of volcanic sand were collected at the foot of the “Mocho-Choshuenco” volcano, of which the last eruption was recorded in 1864 (Rawson et al., 2015). At the other extreme, soil collected at the ESAA, belonging to the Universidad Austral de Chile, corresponds to a developed soil that originated from volcanic ashes (Duric Hapludand; CIREN, 2003) that is locally named Trumao (Valdivia soil Serie). The three volcanic materials were taken to the greenhouse of the Universidad Austral, and sieved through a 5-mm sieve to remove organic material (e.g., roots, leaves, etc.) and other larger debris. These volcanic materials were analyzed chemically in the Soil Laboratory of the Faculty of Agricultural Sciences at the Universidad Austral de Chile, using the methods described in Delgado et al. (2018), showing differences in their nutrient concentrations and other chemical parameters. For example, soil from ESAA presented, on average, a two and six times greater N and P availability, respectively, than the Choshuenco and Ensenada substrates. Likewise, soil from ESAA contained nine and 20 times more total P than Choshuenco and Ensenada substrates, respectively. The youngest volcanic substrate, collected at Ensenada, showed the lowest values of exchangeable cations (Ca^2+^, K^+^, Na^+^, and Mg^2+^) and the highest percentage of Al saturation (Table 1). Additionally, water-retention curves and pore-size distribution were measured for disturbed volcanic materials used in this experiment. For the determination of water-retention curves, saturated samples of each material (230 cm^3^) were drained at decreasing water potential values (0, −6, −15, −33, and −1,500 kPa). The distribution of soil pores was obtained from the water-retention curve as described by Dörner et al. (2010) and, according to pore size classified by Ingram et al. (2015), the lowest amount of plant-available water was found in the youngest volcanic substrate, collected at Ensenada site (Table 1, Supplementary Figure 1).

**TABLE 1:** Chemical and physical analysis of the substrates used in the experiment. Each value corresponds to the average of three soil samples ± standard error (SE).

	**ESAA**	**Choshuenco**	**Ensenada**
N (mg kg^–1^)	45.0 (3.3)	29.4 (6.3)	25.2 (1.4)
P-Olsen (mg kg^–1^)	19.3 (3.2)	2.3 (0.2)	2.4 (0.2)
P total (mg kg^–1^)	1,656 (30)	177 (5.3)	82 (0.2)
pH (H_2_O)	5.59 (0.05)	6.43 (0.2)	6.37 (0.04)
pH (CaCl_2_)	4.87 (0.02)	5.68 (0.2)	5.59 (0.03)
Ca (cmol^+^ kg^–1^)	2.82 (0.3)	0.40 (0.2)	0.1 (0.00)
Mg (cmol^+^ kg^–1^)	0.42 (0.1)	0.09 (0.03)	0.007 (0.00)
K (cmol^+^ kg^–1^)	0.23 (0.01)	0.04 (0.02)	0.004 (0.00)
Na (cmol^+^ kg^–1^)	0.04 (0.02)	0.04 (0.01)	0.001 (0.00)
Al (cmol^+^ kg^–1^)	0.20 (0.00)	0.02 (0.01)	0.03 (0.01)
Sum of cations (cmol^+^ kg^–1^)	3.52 (0.2)	0.65 (0.2)	0.11 (0.00)
ECEC (cmol^+^ kg^–1^)	3.71 (0.2)	0.67 (0.2)	0.14 (0.01)
Al saturation (%)	5.31 (0.3)	4.12 (1.6)	19.8 (2.6)
Bulk density (g cm^–3^)	0.47 (0.2)	1.04 (0.02)	1.03 (0.01)
Total porosity (%)	72.2 (3.6)	58.0 (1.04)	63.8 (0.19)
*Wide pores; >50 μm (%)	18.6 (3.7)	32.9 (0.95)	47.7 (2.07)
**Narrow pores; 50–10 μm (%)	11.0 (0.5)	10.4 (0.24)	5.1 (2.07)
**Middle pores; 10–0.2 μm (%)	26.1 (1.4)	10.4 (0.44)	8.7 (0.3)
***Fine pores; <0.2 μm (%)	16.4 (0.7)	4.4 (0.05)	2.4 (0.01)

ECEC, effective cation-exchange capacity. Function of pore size according to Ingram et al. (2015): *aeration and water transmission, **water-holding capacity, and ^∗∗∗^residual (very strongly bound water and unavailable to plants).

### Plant Material

In March 2016, seeds of *G. avellana*, *E. coccineum*, *L. hirsuta*, *L. ferruginea*, and *L. dentata* were collected from the Botanical Garden of the Universidad Austral de Chile, Valdivia. The seeds were taken to the laboratory (Universidad Austral de Chile, Valdivia) and stored at 4°C for 3 months to perform stratification requirements (Donoso and Escobar, 1986). Then, in order to stimulate germination, seeds were treated with 250 mg L^–1^ of gibberellic acid and placed in a temperature-controlled chamber at 20°C. After 1 month, the germinated seeds were planted in the different volcanic materials described above.

### Experimental Design

Forty-five plants of each species were randomly separated into three groups of 15 seedlings each. Individual plants of each group were planted in 1-L pots of one of the collected materials from each site. The plants were maintained in the greenhouse for 9 months, from July 2016 to April 2017. The average temperature during the experiment was 19.2°C, with maximum and minimum temperatures of 34.8 and 7.2, respectively. The average light intensity was 261 μmol photons m°^2^ s°^1^ between 10:30 and 11:30 AM, with maximum and minimum values of 618 and 72 μmol photons m°^2^ s°^1^, respectively. The plants were irrigated regularly to field capacity with tap water.

### Height and Biomass Determination

At the end of the experiment, the height of the stem was recorded for all seedlings. In addition, seedlings were harvested and separated into leaves, stems, non-cluster roots and CR, and dried in an oven at 60°C for 48 h. Subsequently, the different plant organs were weighed on an analytical balance (AS220-C2 Radwag, Randon, Poland) to determine total biomass and biomass distribution. The number of mature (living) and senesced CR were also determined. For this, the color was used to distinguish between mature (white) and senesced (dark-brown) CR, as described in Delgado et al. (2015a). Additionally, to better understand the nutrient limitation on growth, we used reaction norm approach described by Sadras and Richards (2014), where values of total biomass and total height of each species were expressed in relative terms with respect to total biomass and total height of seedlings grown in the nutrient-poorest substrate.

### Collection and Determination of Root Exudates

Exudates were collected from the total root system of each plant following the methodology described in Delgado et al. (2014). Briefly, the roots were washed with tap water, incubated in CaSO_4_ (0.2 mM) and shaken for 2 h. Subsequently, the solution was filtered, to avoid the presence of microorganisms, with a sterile syringe containing a filter of 0.22 μm. The liquid samples containing the exudates were frozen at −20°C and then lyophilized using a freeze-dryer (Model FD8508, Bondiro, Ilshin Lab, Co. Ltd., Korea). Finally, lyophilized samples were re-suspended in water for high-performance liquid chromatography (HPLC) and quantified using HPLC equipment (JASCO, LC-Net II/ADC, Tokyo, Japan) following the protocol described by Delgado et al. (2013). Citrate, malate, oxalate, and succinate were used as standards. These determinations were carried out at the Institute of Agroindustries of the Universidad de La Frontera, Temuco. The values were expressed as a rate of carboxylates exuded per gram of fresh weight (FW) per hour (μmol g^–1^ FW h^–1^). The exudates from six seedlings per species grown in the different substrates were analyzed.

### Foliar and Seed Mineral Concentrations

Leaves were dried at 60°C in a forced-air oven for 48 h and pulverized to analyze P, N, Mn, Fe, Cu, Zn, and Al concentration. Nitrogen was determined through acid digestion, Kjeldahl distillation and titration (Baker and Thompson, 1992). To determine the other elements, samples were ashed at 500°C for 8 h and then treated with 2 M hydrochloric acid. Phosphorus was determined by colorimetry using the vanadate phosphomolybdate method. Manganese, Fe, Cu, Zn, and Al concentrations were quantified using a simultaneous multielement atomic absorption spectrophotometer (Model 969, Unicam, Cambridge, United Kingdom) using the methodology described by Sadzawka et al. (2004). Additionally, in order to evaluate the influence of seed nutrient content on plant performance, macro- and micronutrients were determined in the seeds of all species. For this, 0.5 g of seeds were milled and the nutrients were determined using the same methodologies described for leaf nutrient concentration. We used the dry weight of the seeds previously reported by Delgado et al. (2014) to determine the nutrient content of the seeds.

### Statistical Analyses

To determine if there were significant differences in the responses of the species and the different volcanic materials, as well as possible interactions between the factors studied, the data were evaluated using a two-way ANOVA with a Tukey’s *a posteriori* test (*P* ≤ 0.05). To determine significant differences in seeds nutrient concentrations and content, we used one-way ANOVA with Tukey’s *a posteriori* test (*P* ≤ 0.05). Additionally, relationships between total plant biomass and nutrient content in seeds were tested by linear regression. ANOVAs and regression analyses were performed using the Sigma Plot v.12 and Graphpad prims v.8, respectively. Finally, a principal component analysis (PCA) was performed to associate chemical variables of the three volcanic materials with the plant traits, using the R Studio program.

## Results

### Height and Biomass Determination

**FIGURE 1:**
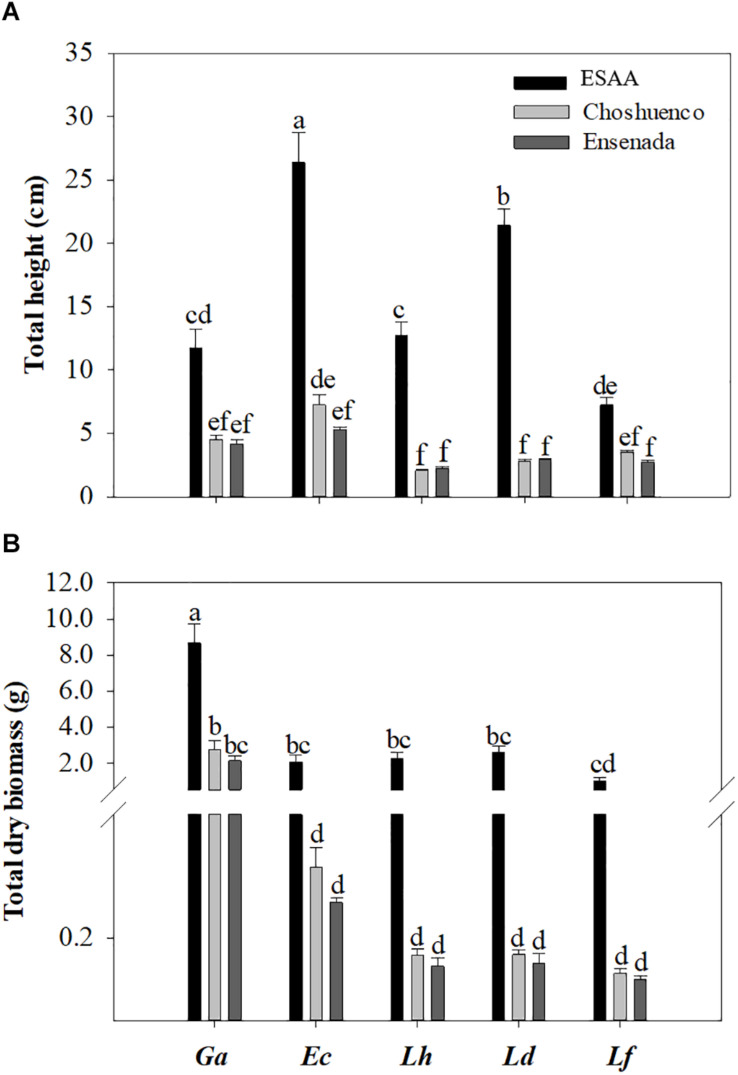
Mean total height **(A)** and total dry biomass **(B)** per plant of *Gevuina avellana* (Ga), *Embothrium coccineum* (Ec), *Lomatia hirsuta* (Lh), *L. dentata* (Ld), and *L. ferruginea* (Lf) grown in three volcanic materials: ESAA, Choshuenco and Ensenada. Each bar corresponds to the average per plant (*n* = 15) ± standard error (SE). Different letters indicate significant differences among species and volcanic materials (*P* ≤ 0.05).

All species showed the best performance, i.e., total height and total dry biomass, when grown in ESAA soil (Figures 1A,B). For seedlings grown in ESAA soil, the total height and total dry biomass were about six to eight times and three to 19 times greater than those of the seedlings grown on Choshuenco and Ensenada substrates, respectively (Supplementary Figure 3). The growth response varied significantly (*P* ≤ 0.05) among species, even on the same substrate. Thus, in ESAA soil, *E. coccineum* and *L. dentata* seedlings showed the greatest growth in total height, being significantly greater than those in *G. avellana*, *L. hirsuta*, and *L. ferruginea* seedlings (Figure 1A). However, the greatest total dry biomass was found in *G. avellana* which was significantly (*P* ≤ 0.05) greater than that in the other species (Figure 1B). On the poorest substrates, Choshuenco and Ensenada, *G. avellana* was also the species showing the greatest total dry biomass, followed by *E. coccineum* and the *Lomatia* species. In general, we observed no significant (*P* ≤ 0.05) differences in total height and total dry biomass of the species grown on the Ensenada and Chohuenco substrates.

**FIGURE 2:**
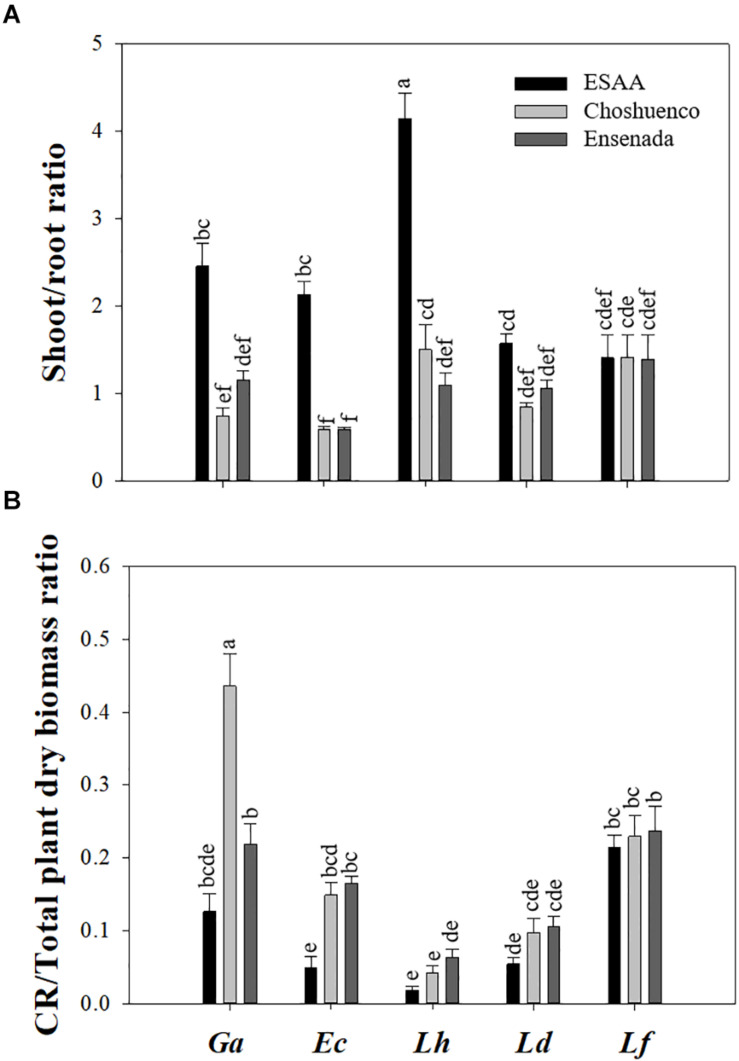
Shoot/root ratio **(A)** and cluster roots (CR)/total plant dry biomass ratio **(B)** of *Gevuina avellana* (Ga), *Embothrium coccineum* (Ec), *Lomatia hirsuta* (Lh), *L. dentata* (Ld), and *L. ferruginea* (Lf) grown in three volcanic materials: ESAA, Choshuenco and Ensenada. Each bar corresponds to the average per plant (*n* = 15) ± standard error (SE). Different letters indicate significant differences among species and volcanic materials (*P* ≤ 0.05).

Except for *L. ferruginea*, shoot/root ratio was significantly (*P* ≤ 0.05) affected by volcanic material. Thus, the highest shoot/root ratio values were found in the plants grown in soil from ESAA compared with those grown on substrates from Ensenada and Chohuenco (Figure 2A). Similarly, the CR/Total plant dry biomass ratio was also significantly affected by substrate, with higher values on the poorest substrates, Choshuenco and Ensenada (Figure 2B). Interestingly, *G. avellana* showed a significantly (*P* ≤ 0.05) higher CR/Total plant dry biomass ratio on Choshuenco substrate than on the other two substrates (Figure 2B).

### Number and Biomass of Cluster Roots (CR) and Carboxylate Exudation Rate From Whole Root Systems

**FIGURE 3:**
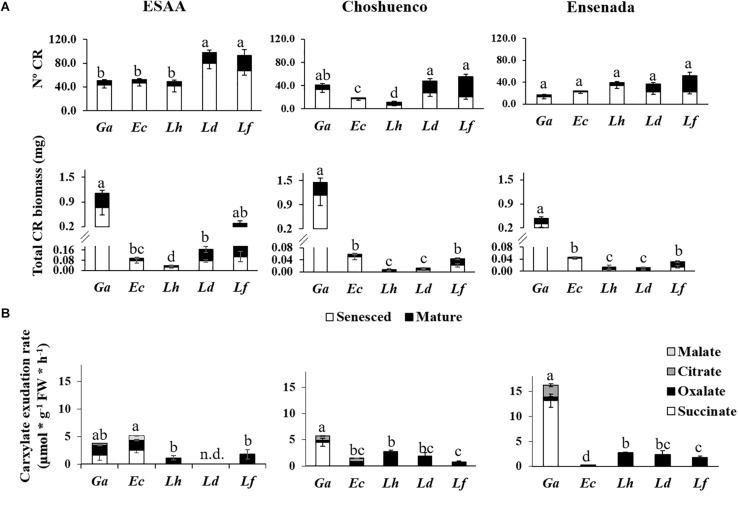
**(A)** Mean number and biomass of senesced and mature cluster roots (CR) per plant (*n* = 15) and **(B)** carboxylate-exudation rates from entire root systems (*n* = 6) of *Gevuina avellana* (Ga), *Embothrium coccineum* (Ec), *Lomatia hirsuta* (Lh), *L. dentata* (Ld), and *L. ferruginea* (Lf) grown in three volcanic materials: ESAA, Choshuenco and Ensenada. Different letters in **(A)** indicate significant differences in total (sum of mature and senesced) number and biomass of CR among species on the same volcanic material (*P* ≤ 0.05). Different letters in **(B)** indicate significant differences in total (sum of carboxylates) carboxylate exudation rate among species on the same volcanic material (*P* ≤ 0.05).

All seedlings produced CR, even those grown in the soil richest in nutrients (ESAA). In fact, a greater average number and biomass of CR was observed when the seedlings were grown in the richest soil than in the nutrient-poor substrates, Choshuenco and Ensenada (Figure 3A). In ESAA soil, *L. dentata* and *L. ferruginea* were the species that presented the largest number of CR. However, their biomass was similar or less than that of the other species, especially in the nutrient-poor substrates. Conversely, *G. avellana* was the species with the greatest CR biomass in all volcanic materials, and it also exhibited the fastest carboxylate-exudation rate, especially on the nutrient-poor substrates (Figure 3B). The main carboxylate exuded by roots of *G. avellana* was succinate, whereas *Lomatia* species exuded only oxalate. *Embothrium coccineum* had similar or more CR biomass than *Lomatia* species, but exuded carboxylates at a slower rate than these species (Figure 3A).

### Foliar Nutrient Concentrations

**FIGURE 4:**
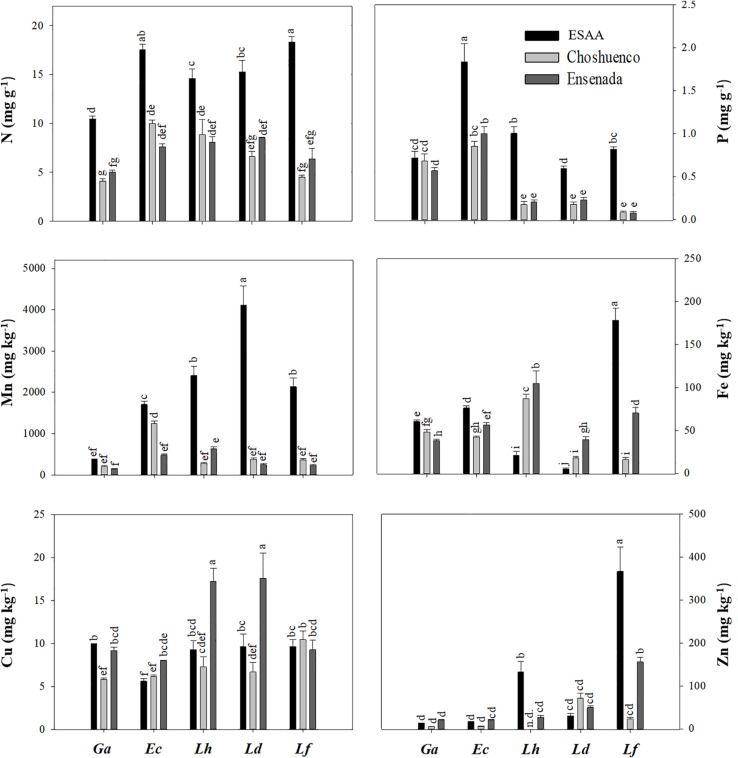
Foliar nitrogen (N), phosphorus (P), manganese (Mn), iron (Fe), copper (Cu), and zinc (Zn) concentrations in *Gevuina avellana* (Ga), *Embothrium coccineum* (Ec), *Lomatia hirsuta* (Lh), *L. dentata* (Ld), and *L. ferruginea* (Lf) grown in three volcanic materials: ESAA, Choshuenco, and Ensenada. Each bar corresponds to the average per plant (*n* = 6) ± standard error (SE). Different letters indicate significant differences among species and volcanic materials (*P* ≤ 0.05).

**FIGURE 5:**
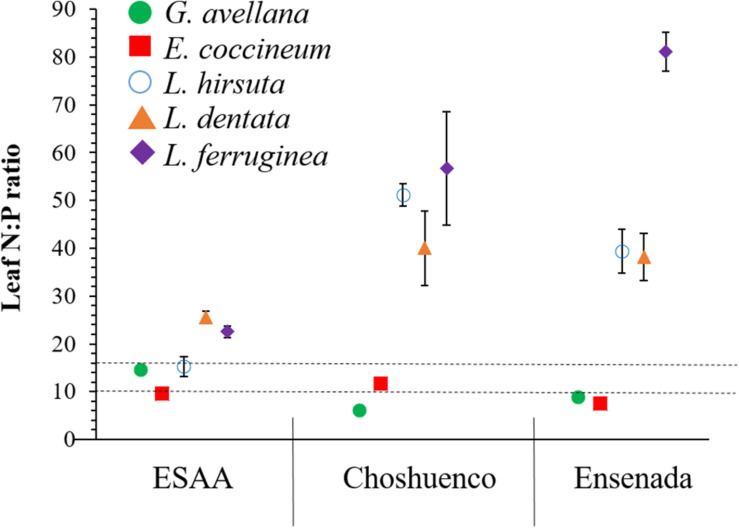
Leaf nitrogen:phosphorus (N:P) ratio (*n* = 6) in *Gevuina avellana*, *Embothrium coccineum*, *Lomatia hirsuta*, *L. dentata*, and *L. ferruginea* grown in three volcanic materials: ESAA, Choshuenco, and Ensenada. Horizontal lines indicate N limitation (values < 10), *P* limitation (values > 16) or both N and P limitation (values 10–16) according to Koerselman and Meuleman (1996).

Foliar nutrient concentrations varied significantly (*P* ≤ 0.05) among species and volcanic material they were grown in (Figure 4). Due to the fact that the mineral N concentration in the Choshuenco and Ensenada substrates is about half that in the ESAA soil (Table 1), we found that all species grown in nutrient-poor substrates showed significantly (*P* ≤ 0.05) lower leaf N concentrations than when grown in ESAA soil (Figure 4). In addition, leaf P concentrations were also significantly (*P* ≤ 0.05) higher in plants grown in ESAA soil (Figure 4). Interestingly, the only exception was *G. avellana*, which had similar foliar P concentrations in the three volcanic materials, independent of the basal P concentration in them. *Embothrium coccineum* was the species showing the significantly (*P* ≤ 0.05) highest leaf P concentration when grown in the ESSA soil. Similarly, this species along with *G. avellana*, presented significantly (*P* ≤ 0.05) higher leaf P concentrations than the *Lomatia* species when grown in Choshuenco and Ensenada substrates. In these nutrient-poor volcanic substrates, *L. hirsuta* and *L. dentata* showed similar foliar P concentrations, but significantly (*P* ≤ 0.05) higher values than those found in *L. ferruginea* (Figure 4). Interestingly, leaf N:P ratios in *Lomatia* species was greater than 16 when grown on the poorest substrate, in contrast to *G. avellana* and *E. coccineum*, in which it was around 10 (Figure 5).

In general, the plants that grew on the ESAA soil presented higher values of leaf Mn concentration than those on the Choshuenco and Ensenada substrates. *Lomatia dentata* was the species that had the highest Mn concentration in its leaves, while *G. avellana* was the species that had the lowest leaf Mn concentration on all substrates (Figure 4).

Except for *L. ferruginea*, leaf Cu concentration varied significantly (*P* ≤ 0.05) among the volcanic materials. In general, plants grown on Ensenada substrate showed the highest values of leaf Cu concentration, especially in *L. hirsuta* and *L. dentata* (Figure 4). *Lomatia ferruginea* was the species with the highest leaf concentrations of Fe and Zn, especially on ESAA soil and Ensenada substrate. In the other species, the leaf Fe and Zn concentration was variable and depending on the volcanic material. For example, the leaf Fe concentrations in *G. avellana* and *E. coccineum* were higher in ESAA soil, while in *L. hirsuta* and *L. dentata* the concentrations were higher in the nutrient-poor substrates, Choshuenco and Ensenada. With respect to leaf Zn concentration, the general tendency was to find lower values in plants grown in the Choshueco substrate, except for *L. dentata*, where a higher leaf Zn concentration was found than in plants grown in ESAA soil and Ensenada substrate.

### Seed Nutrient Concentrations and Contents

*Gevuina avellana* had the lowest macro- and micronutrient concentrations in its seeds compared with the other Proteaceae (Supplementary Table 1). However, due to the larger size of its seeds, the total nutrient content was significantly (*P* ≤ 0.05) greater (Table 2). Thus, the values of N and P content were, on average, nine times greater in *G. avellana* than in *E. coccineum*, while, values of N and P content were on average 26 and 17 times greater in *G. avellana* than in *Lomatia* species, respectively. The micronutrient contents varied among species, but in general the trend was that *G. avellana* had the highest micronutrient content, followed by *E. coccineum* and then *Lomatia* species. The regression analyses revealed a relationship between total plant biomass and nutrient content in seeds (Supplementary Figure 4).

**TABLE 2:** Nitrogen (N), phosphorus (P), manganese (Mn), iron (Fe), copper (Cu), zinc (Zn), and aluminum (Al) content in seeds of *Gevuina avellana*, *Embothrium coccineum*, *Lomatia hirsuta*, *L. dentata*, and *L. ferruginea*.

**Species**	***G. avellana***	***E. coccineum***	***L. hirsuta***	***L. dentata***	***L. ferruginea***
N (μg seed^–1^)	4,482 (372)^*a*^	486 (9.0)^*b*^	185 (20)^*c*^	152 (8.5)^*c*^	182 (7.2)^*c*^
P (μg seed^–1^)	642 (28)^*a*^	71 (2.6)^*b*^	30.2 (1.1)^*e*^	37 (2.0)^*d*^	47 (1.7)^*c*^
Mn (μg seed^–1^)	8.6 (1.0)^*a*^	4.86 (0.2)^*b*^	2.06 (0.1)^*c*^	5.90 (0.1)^*a,b*^	2.45 (0.1)^*c*^
Fe (μg seed^–1^)	12.8 (1.5)^*a*^	0.82 (0.02)^*b*^	0.48 (0.02)^*c*^	0.31 (0.01)^*d*^	0.52 (0.02)^*c*^
Zn (μg seed^–1^)	5.0 (0.3)^*a*^	0.81 (0.04)^*b*^	0.24 (0.00)^*e*^	0.28 (0.00)^*d*^	0.33 (0.00)^*c*^
Cu (μg seed^–1^)	2.6 (0.2)^*a*^	0.20 (0.00)^*b*^	0.08 (0.00)^*d*^	0.07 (0.00)^*e*^	0.10 (0.00)^*c*^
Al (μg seed^–1^)	1,371 (122)^*a*^	1.22 (0.01)^*b*^	0.09 (0.06)^*d*^	0.34 (0.03)^*c*^	0.32 (0.03)^*c*^

*The nutrient and Al content data were calculated using the dry weight of seeds of each species determined by Delgado et al. (2014). Each value corresponds to the average of three batches of seed ± standard error (SE). Different letters indicate significant differences among species (*P* ≤ 0.05).*

### Volcanic Materials and Plant Traits

**FIGURE 6:**
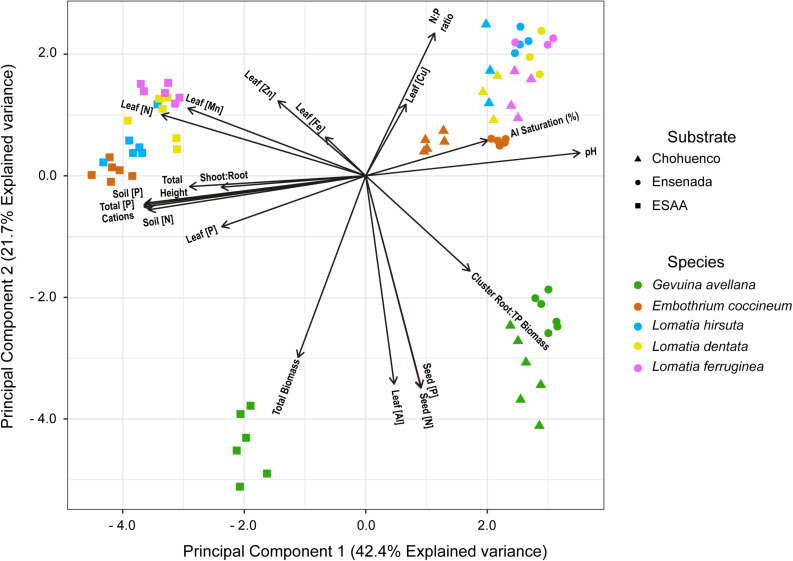
Principal component analysis representing different plant traits of five southern South American Proteaceae [e.g., leaf nitrogen (N), phosphorus (P), manganese (Mn), iron (Fe), zinc (Zn), copper (Cu), and aluminum (Al) concentrations (mg g^– 1^), leaf N:P ratio, seed nitrogen (N) and phosphorus (P) concentration, total height, total biomass, shoot:root ratio, cluster root:total plant biomass ratio (cluster root: TP biomass)] and soil variables [e.g., soil mineral N concentration (soil (N), mg kg^– 1^), available P soil (soil P, mg kg^– 1^), soil pH (pH), sum of cations (cations), Al saturation (%) and total P (mg kg^– 1^)] of three volcanic materials (ESAA, Choshuenco, and Ensenada) where these species were grown.

From the PCA performed on volcanic materials and plant traits, we found clear separations across the horizontal axe between groups of plants that were grown on the nutrient-rich volcanic soil (EEAA) versus those grown on young and nutrient-poor volcanic substrates (Choshuenco and Ensenada) (Figure 6). Likely, species grown on EEAA were joined because they presented, in general, higher leaf macronutrient (N and P) concentrations, total height and shoot:root ratio than those grown on Choshuenco and Ensenada substrates. In addition, among species grown on young and nutrient-poor volcanic substrates, *E. coccineum* was separated from *Lomatia* species, mainly explained by their differences in the foliar N:P ratio. Finally, the PCA also revealed that *G. avellana* plants were separated from the rest of the species (lower quadrant), probably because it presented the highest N and P content in the seeds, leaf Al concentration, total biomass and CR:total plant biomass ratio (Figure 6).

## Discussion

In general, *G. avellana* showed greater total dry biomass and *E. coccineum* presented greater height in all substrates compared with the other species (Figures 1A,B). Most likely, the reason why *G. avellana*, in its early stages, produced more biomass than the other Proteaceae species is its larger seeds (Delgado et al., 2014) and higher nutrient content compared with those of the other species (Table 2), favoring its initial growth (Supplementary Figure 4). This trend was maintained in *E. coccineum*, which is the species that has the second-largest seed nutrient content (Table 2). Even though there is clear evidence that seed size and nutrient content play an important role in early stablisment of species on nutrient-poor soils (Milberg and Lamont, 1997; Denton et al., 2007), the greater growth of *G. avellana* and *E. coccineum* in poor soils cannot be explained by the nutrient content in their seeds alone, since in the case of *G. avellana*, almost all the plants (88%) had shed their cotyledons 2 months after starting the experiment (data not shown). Thus, these plants must have additional strategies to sustain growth and development under nutrient deprivation. For example, plants tend to allocate a greater proportion to root biomass and thus increase the chance to acquire nutrients (Bloom et al., 1985; Chapin et al., 1987; Gedroc et al., 1996). Our study supports this idea, because shoot/root ratios of all species (with the exception of *L. ferruginea*) were significantly lower in plants grown on the poorest substrates than on EEAA soil (Figure 2A). Likewise, the CR/total plant dry biomass ratio was higher in plants grown on recent volcanic substrates than in those grown in the nutrient-rich soil (Figure 2B), showing that, as reported in *Hakea prostrata* R. Br. (Proteaceae) (Shane et al., 2003), the biomass allocation to CR is greater when plants grow under nutrient deprivation. Interestingly, *L. ferruginea* was the only species that did not adjust its relative biomass distribution (e.g., shoot/root ratio, CR/total plant dry biomass ratio) in response to nutrient availability, suggesting a constitutive biomass-distribution pattern in this species.

In combination with root morphological traits as a strategy to explore the soil for nutrients, species bearing CR modify their metabolism and enhance the biosynthesis and release of carboxylates (Neumann et al., 1999; Shane et al., 2004, 2013), which play an important role in nutrient mobilization from the soil. Carboxylate composition of root exudates depends on the soil/substrate and species (Lambers et al., 2002; Veneklaas et al., 2003; Shi et al., 2019). In our study, a mixture of carboxylates (succinate, malate, citrate and malate) was found in root exudates of *G. avellana* and *E. coccineum*, while in *Lomatia* species only oxalate was detected (Figure 3B). Succinate was the main carboxylate exuded by the roots of *G. avellana*, which is the species with the highest total CR biomass (Figures 3A,B). This carboxylate is unusual in other species bearing CR, where citrate or malate are usually the major carboxylates exuded (Roelofs et al., 2001; Shane et al., 2004; Delgado et al., 2014; Ávila-Valdés et al., 2019), although it has been detected in the root exudates of *Medicago sativa* L. seedlings (Lipton et al., 1987) and two *Phaseolus vulgaris* L. genotypes (Atemkeng et al., 2011) when plants were subjected to low-P stress. Interestingly, *Lomatia* species only exuded oxalate. Zúñiga-Feest et al. (2020) reported similar results in root exudates of *L. dentata*. These authors suggest that oxalate could play an important role in P mobilization, because this organic anion has effects similar to citrate in P mobilization (Gerke et al., 2000). On the other hand, *E. coccineum* had more CR biomass than *Lomatia* species, but exuded carboxylates at a slower rate than *Lomatia* species, probably because at the time of the plant harvest (autumn), most CR had senesced in *E. coccineum*, as reported by Donoso-Ñanculao et al. (2010). These authors observed that there is a seasonal variation in CR formation in *E. coccineum*, with a greater proportion of mature CR relative to total root biomass in spring than in autumn. Seasonal variation of CR formation in *Lomatia* species has not been assessed, but apparently these species can maintain or produce mature CR for longer than the other species, especially *L. dentata* and *L. ferruginea* (Figure 3A). Although in the present study *E. coccineum* did not exude large amounts of carboxylates, Delgado et al. (2014) reported that CR of *E. coccineum* can exude large amounts of citrate and malate, even more than CR of *H. prostrata* (Shane et al., 2004), a species that occurs in extremely nutrient-poor soils, when grown in similar hydroponic conditions. Therefore, the composition and quantity of exudates depend on the time exudates are collected and on experimental conditions.

In order to avoid misinterpretation of the roots exudates collected in a single moment, some authors have suggested using other techniques as a proxy of the cumulative effect of carboxylates. Thus, leaf Mn concentration can be used as a proxy for carboxylate exudation or P-acquisition potential (Shane and Lambers, 2005b; Hayes et al., 2014; Lambers et al., 2015b, 2021; Pang et al., 2018; Shi et al., 2019). This is because carboxylates exuded by roots simultaneously mobilize P and other nutrients from the rhizosphere, especially Mn. The measurement of leaf Mn concentration is a valuable indicator of the cumulative effect of carboxylate exudation (Shane and Lambers, 2005b; Pang et al., 2018). Our results show that *Lomatia* species grown on the ESAA soil, had higher leaf Mn concentrations than *G. avellana* and *E. coccineum* (Figure 4), indicating that *Lomatia* roots released more carboxylates when grown in a nutrient-rich soil. These results are consistent with the fact that *L. dentata* and *L. ferruginea* produced a greater number and more biomass of CR (Figure 3A) when plants were grown on ESAA soil. Therefore, our findings suggest that the non-colonizing species, *L. dentata* and *L. ferruginea*, when grown on nutrient-richer soils responded rapidly forming CR and, presumably, maintained rapid rates of carboxylate exudation over time, as evidenced by their high leaf Mn concentration. Among Proteaceae that grew in the young and nutrient-poor substrates, *E. coccineum* was the species that presented the highest leaf Mn concentration. This species is commonly found colonizing volcanic substrates/soils (Donoso, 2006) which might be explained by its high capacity to exude carboxylates (in this study supported by its high leaf Mn concentration). In contrast, *Gevuina avellana* is a species that also can colonize and thrive on recent volcanic substrates. However, the relatively low Mn concentration in its leaves (Figure 4) suggests a relatively low carboxylate-exudation capacity. Recently, Delgado et al. (2019) reported that *G. avellana* hyperaccumulates aluminum (Al) in its leaves. That study was carried out at various sites along the natural geographical distribution of Proteaceae species (37.23°–51.22°S), and showed that leaf Mn concentration of *G. avellana* was much lower than that of non-Al hyperaccumulator species of the Proteaceae family from the same region. A similar trend was found in the present study, where *G. avellana* plants hyperaccumulated Al in its leaves (Supplementary Figure 2), but had the lowest leaf Mn concentrations compared with the other species (Figure 4). In this context, several studies have shown negative correlations between Mn and Al concentrations in leaves (Foy et al., 1973; Blair and Taylor, 1997; Fernando et al., 2009). However, the antagonistic uptake of these two metal ions remains unclear, because the two metals are taken up by different transport systems (Nevo and Nelson, 2006; Xia et al., 2010).

Interestingly, the Proteaceae species we studied differed in their leaf nutrient concentrations. For example, *G. avellana* was the species with the lowest leaf N concentration, especially in ESAA soil. This might be related to its nutrient-conservation strategy, since species with long leaf lifespan, such as *G. avellana* (4.3–5.4 years; Lusk and Corcuera (2011), tend to produce thicker leaves (Wright et al., 2004) with low N concentration on a weight basis (Reich et al., 1991; Reich et al., 1998), which enhances their robustness and decreases their palatability. Another trait revealing its nutrient-conservation strategy is the similar P concentrations found in leaves of *G. avellana* seedlings grown on the three volcanic materials evaluated (Figure 4). These results suggest that this species has a low P requirement and tightly down-regulates its P-uptake capacity when grow in soil with higher P availability (ESAA). Similar results have been found in other species bearing CR (e.g., *Viminaria juncea* (Schrad.) Hoffmanns (Fabaceae) (De Campos et al., 2013b), *Euplassa cantareirae* Sleumer (Proteaceae) (De Britto Costa et al., 2015), and this trait might be related to the ability of those species to avoid toxicity caused by excess soil P (Shane and Lambers, 2005c; De Campos et al., 2013a). We suggest that this ability allows *G. avellana* to develop well in a wide range of soil conditions, from deep soils with high fertility to volcanic substrates such as lava and slag (Donoso, 2006; Delgado et al., 2018). Some of the other studied Proteaceae can also grow in a wide range of soil conditions, but they probably use the P to grow faster when they occur in more fertile soils. Thus, *G. avellana* was the species that presented minor changes (four-fold) in plant biomass when grown in recent volcanic substrates versus nutrient-rich soil. Conversely, *E. coccineum*, *L. ferruginea*, *L. hirsuta*, and *L. dentata* produced, on average, up to 7, 10, 16, and 18 times more biomass, respectively, in fertile soil than in recent volcanic substrates (Figure 1B, Supplementary Figure 3). Zúñiga-Feest et al. (2020) also found that *G. avellana* has a more conservative relative growth rate when it grows in sand watered with different nutrient concentrations (full nutrient solution, without P, without N, water), while *L. dentata* grows faster when watered with complete nutrient solution. These results support the idea that *Lomatia* species maximize their growth under nutrient-rich soils, probably to compete in more fertile and diverse plant communities.

On the poorest substrates, Choshuenco and Ensenada, the leaf N:P ratio in *Lomatia* species was greater than 16, whereas in *G. avellana* and *E. coccineum* the N:P ratio was less than or close to 10 (Figure 5). According to the N:P ratios for vegetation representing the nature of nutrient limitation (Koerselman and Meuleman, 1996), our finding indicate P limitation in the leaves of *Lomatia* species and N limitation in the leaves of *G. avellana* and *E. coccineum*. These results contrast with those previously reported by Delgado et al. (2018), who determined the N:P ratios in the same species of this study, finding that adult plants growing in a wide variety of climatic and edaphic conditions are mainly limited by P. We postulate that newly emerged seedlings of *Lomatia* species have higher P requirements for triggering CR formation than *G. avellana* and *E. coccineum*. These results were more evident for *L. dentata* and *L. ferruginea*, which were limited by P even in the nutrient-richest soil (Figure 5), where they also produced more total CR biomass (Figure 3A). Alternatively, these results suggest that CR of *E. coccineum* and *G. avellana* could be more effective at acquiring P than the CR of *Lomatia* species which is evidenced by the fact that leaves of *G. avellana* and *E. coccineum* showed the highest leaf P concentration, even on the poorest substrates.

With respect to micronutrient concentrations, these were highly variable depending on species and volcanic material, especially in *Lomatia* species, which presented greater differences in their foliar copper (*L. hirsuta* and *L. dentata*) and zinc (*L. ferruginea*) concentrations than *G. avellana* and *E. coccineum*. Additionally, *L. ferruginea* showed the widest range of foliar iron (Fe) concentrations, reaching the highest Fe concentrations when grown on ESAA soil. This is consistent with Delgado et al. (2019), who found a wide variation in leaf Fe concentration under natural conditions. According to Delgado et al. (2019), this high variation in leaf Fe concentration is not correlated with soil Fe availability, and, therefore, it would be interesting to study the factors that influence the different Fe-uptake rates in these species, which apparently show the same trend as those of zinc uptake (Figure 4).

In summary, our study reveals that, although Proteaceae is a family widely known to produce carboxylate-releasing CR (Lambers et al., 2021), there are great differences among species in relation to their ability to thrive on soil/substrates with different nutrient availability (Figure 6). Probably, species that are better adapted to grow on relatively more fertile soils (e.g., *Lomatia* species), have decreased and/or lost the functionality of their CR when they grow in extremely nutrient-poor soils, perhaps because they are not able to recover the costs associated with the formation and functioning of these root structures. In fact, it has recently been reported that *Xylomelum occidentale*, a Proteaceae growing on soil that is moderately less P-impoverished than those in representative Proteaceae habitats in south-western Australia, do not produce functional CR (Zhong et al., 2021). In this context, it is necessary to mention that the better performance of these species under different soil nutrient conditions cannot be fully explained by a single trait. In fact, the plant adaptations to certain environmental conditions involve a complex network of physiological, biochemical and molecular responses, which, until now, are far from being fully understood in native plants. In our study, we contributed to the understanding of the autoecology of southern South American Proteaceae through the identification of some traits, such as seed nutrient content, nutrient requirements and ability to mobilize nutrients, that help us to explain – at least in part – the differential colonization capacities and performance under field conditions of these species.

## Conclusion

We conclude that Proteaceae species vary widely in their ability to grow and acquire nutrients in young and nutrient-poor volcanic substrates. *Gevuina avellana* and *E. coccineum* performed better on young nutrient-poor volcanic substrates than *Lomatia* species. On the one hand, the seedling growth of larger-seeded *G. avellana* was less affected by nutrient availability of the soil/substrate, probably because it had a greater nutrient content in its seeds and produced CR exuding a large amount of carboxylates that supported their initial growth. On the other hand, *E. coccineum* exhibited greater total plant height and leaf P concentration than *Lomatia* species, presumably due to greater carboxylate exudation over time, as evidenced by their higher leaf Mn concentration (used as a proxy for carboxylate exudation) in one of the nutrient-poor volcanic substrates. Understanding the ecophysiology and functioning of these species in nutrient-poor soils can provide valuable tools to be used in restoration with these native species or other species with similar traits.

## Data Availability Statement

The original contributions presented in the study are included in the article/Supplementary Material, further inquiries can be directed to the corresponding author/s.

## Author Contributions

MD contributed to conceptualization, design of methodology, organization (i.e., graphs and tables), and interpretation of the data, along with writing the original draft. AZ-F contributed in the conception of this study and contributed with important intellectual content at all stages. MR-D contributed to perform chemical analysis of leaf samples as well as carboxylates determination. PB contributed to writing the original draft. SR contributed in the setup, supervision and harvest of the experiment. AB-B contributed to soil/substrates collection in the field as well as in the setup, supervision and harvest of the experiment. SV contributed to chemical analysis of soil and seed samples as well as statistical analysis. MP contributed to soil physical analysis, design of figures, and important intellectual content at the final stage. HL contributed to writing and editing of the manuscript. All authors revised critically the manuscript and approved the final version.

## Conflict of Interest

The authors declare that the research was conducted in the absence of any commercial or financial relationships that could be construed as a potential conflict of interest.
