# Supplementary material for: Ecophysiological Performance of Proteaceae Species From Southern South America Growing on Substrates Derived From Young Volcanic Materials
Source: Front Plant Sci. 2021 Feb 19;12:636056. doi: 10.3389/fpls.2021.636056 (PMC7933449; doi:10.3389/fpls.2021.636056)
Supplement: Supplementary file 1 [file Data_Sheet_1.docx]

**Table Supplementary 1.** Nitrogen (N), phosphorus (P), manganese (Mn), iron (Fe), copper (Cu), zinc (Zn) and aluminum (Al) concentration in seeds of *Gevuina avellana*, *Embothrium coccineum*, *Lomatia hirsuta*, *L. dentata* and *L. ferruginea*. Each value corresponds to the average of three batches of seed ± standard error (SE). Different letters indicate significant differences among species (*P* ≤ 0.05).

| Species | *G. avellana* |  | *E. coccineum* |  | *L. hirsuta* |  | *L. dentata* |  | *L. ferruginea* |
| --- | --- | --- | --- | --- | --- | --- | --- | --- | --- |
| N (mg g^-1^) | 12.7 (1.1) d |  | 28.2 (0.5) b |  | 35.0 (3.8) a |  | 24.1 (1.3) b |  | 20.7 (0.9) c |
| P (mg g^-1^) | 1.8 (0.1) c |  | 4.1 (0.2) b |  | 5.7 (0.2) a |  | 5.9 (0.3) a |  | 5.4 (0.2) a |
| Mn (mg kg^-1^) | 24 (2.7) d |  | 282 (13.6) c |  | 387 (9.8) b |  | 936 (12.0) a |  | 277 (16.2) c |
| Fe (mg kg^-1^) | 36.0 (4.3) c |  | 47.8 (0.9) bc |  | 89.7 (3.4) a |  | 49.8 (2.1) b |  | 59.1 (2.5) b |
| Zn (mg kg^-1^) | 14.2 (0.8) d |  | 47.1 (2.3) a |  | 45.5 (0.04) ab |  | 44.0 (0.5) ab |  | 37.7 (0.5) c |
| Cu (mg kg^-1^) | 7.3 (0.5) c |  | 11.6 (0.2) b |  | 15.0 (0.4) a |  | 11.5 (0.5) b |  | 11.9 (0.3) b |
| Al (mg kg^-1^) | 3872 (346) a |  | 70.8 (0.5) b |  | 16.7 (10.7) d |  | 54.5 (4.9) c |  | 35.9 (3.5) c |

**Supplementary Figure 1.** Water-content (% of volumen) of three volcanic materials used in the experiment: ESAA, Choshuenco and Ensenada. Each value corresponds to the average of four samples ± standard error (SE). The samples were taken from disturbed volcanic materials, similar to those used in the pots of this experiment .

**Supplementary Figure 2.** Foliar aluminum (Al) concentration in *Gevuina avellana* (Ga), *Embothrium coccineum* (Ec), *Lomatia hirsuta* (Lh), *L. dentata* (Ld) and *L. ferruginea* (Lf) grown in three volcanic materials: ESAA, Choshuenco and Ensenada. Each bar corresponds to the average per plant (n = 6) ± standard error (SE). Different capital letters indicate significant differences among species on the same volcanic material and different lower-case letters indicate differences among volcanic materials within the same species (*P* ≤ 0.05).

**Supplementary Figure 3**. Reaction norms for the relative variation of different traits assessed in *Gevuina avellana* (*Ga*), *Embothrium coccineum* (*Ec*), *Lomatia hirsuta* (*Lh*), *L. dentata* (*Ld*) and *L. ferruginea* (*Lf*) grown in three volcanic materials: ESAA, Choshuenco and Ensenada. Values of total biomass and total height correspond to mean response of each specie (n=15) grown in ESAA and Choshuenco substrate and were expressed in relative terms with respect to mean values found for de same specie grown in Ensenada substrate.

**Supplementary Figure 4.** Total biomass per plant as a function of the total P and N contend in seeds of five Proteaceae species: *Gevuina avellana*, *Embothrium coccineum*, *Lomatia hirsuta*, *L. dentata* and *L. ferruginea* grown in three volcanic materials: ESAA, Choshuenco and Ensenada. * indicate that slope of linear model fitted was significatively different from 0.
